# Non-protein nitrogen supplementation on in vitro fermentation profile, methane production, and microbial nitrogen synthesis in a corn silage-based substrate

**DOI:** 10.1093/tas/txae065

**Published:** 2024-04-23

**Authors:** Juan de J Vargas, Federico Tarnonsky, Federico Podversich, Araceli Maderal, Ignacio Fernández-Marenchino, Wilmer Cuervo, Tessa M Schulmeister, Isabel Ruiz-Ascacibar, Ignacio R Ipharraguerre, Nicolás DiLorenzo

**Affiliations:** Department of Animal Sciences, North Florida Research and Education Center, University of Florida, Marianna, FL 32446, USA; Department of Animal Sciences, North Florida Research and Education Center, University of Florida, Marianna, FL 32446, USA; Department of Animal Sciences, North Florida Research and Education Center, University of Florida, Marianna, FL 32446, USA; Department of Animal Sciences, North Florida Research and Education Center, University of Florida, Marianna, FL 32446, USA; Department of Animal Sciences, North Florida Research and Education Center, University of Florida, Marianna, FL 32446, USA; Department of Animal Sciences, North Florida Research and Education Center, University of Florida, Marianna, FL 32446, USA; Department of Animal Sciences, North Florida Research and Education Center, University of Florida, Marianna, FL 32446, USA; Yara Industrial Solutions, Madrid, Spain; Institute of Human Nutrition and Food Science, Christian-Albrechts-University, Kiel, Germany; Department of Animal Sciences, North Florida Research and Education Center, University of Florida, Marianna, FL 32446, USA

**Keywords:** backgrounding diets, biuret, nitrates, non-protein nitrogen, urea

## Abstract

Non-protein nitrogen (**NPN**) supplements improve animal performance in backgrounding diets. However, there is scarce information regarding the effect of different NPN sources and combinations on ruminal fermentation profile. The current study aimed to evaluate the effect of different NPN sources and their combinations on in vitro fermentation, microbial N synthesis, and methane (CH_4_) production in a backgrounding diet. Incubations were conducted on three separate days for 24 h using corn silage and cotton gin byproduct (70% and 30% of DM, respectively) as substrate. Treatments were control (without NPN), urea, and five different proportions of urea–biuret and nitrate (100:0, 75:25, 50:50, 25:75, and 0:100). Each treatment, except control, was formulated to be isonitrogenous and equivalent to 1% urea inclusion. Ruminal fluid was collected from two ruminally cannulated Angus crossbred steers fed ad libitum corn silage and cotton gin byproduct plus 100 g of a urea–biuret–nitrate mixture. The concentration of volatile fatty acids (**VFAs**) and ammonia nitrogen (**NH**_**3**_**-N**) were determined at 12 and 24 h of incubation. Final pH, in vitro dry and organic matter digestibility, total gas production, and concentration of CH_4_ were determined at 24 h. The supplementation of NPN increased (*P* < 0.05) the concentration of NH_3_-N at 12 and 24 h. Although NPN supplementation increased (*P* < 0.05) the concentration of total VFA and acetate at 12 h, treatments did not differ (*P* > 0.05) at 24 h. Supplementation of NPN increased (*P* < 0.05) the proportion of acetate at 12 and 24 h but tended to reduce (*P* = 0.054) the proportion of propionate only at 12 h. Digestibility and pH were not different (*P* > 0.05) among treatments. Increasing nitrates in the NPN supplement increased (*P* < 0.05) the proportion of acetate and reduced (*P* < 0.05) the proportion of butyrate at 12 and 24 h. The supplementation of NPN increased (*P* < 0.05) microbial N synthesis. Furthermore, increasing nitrate proportion in the NPN supplement increased (*P* < 0.05) the microbial N synthesis and efficiency of N use. Supplementation of NPN did not modify (*P* > 0.05) total gas or CH_4_ production. However, increasing nitrate proportion in the NPN supplement linearly reduced (*P* < 0.05) CH_4_ production. Supplementation of NPN increased NH_3_-N concentration and microbial N while increasing the inclusion of nitrate decreased the production of CH_4_ and increased the microbial N synthesis in a corn silage-based substrate under in vitro conditions.

## Teaser

Limited information is available regarding the effect of non-protein nitrogen (NPN) mixtures on fermentation and methane emissions. The inclusion of NPN sources modifies the fermentation increasing microbial synthesis and reducing methane under in vitro conditions.

## INTRODUCTION

Non-protein nitrogen (**NPN**) is a valuable resource in ruminant nutrition because it can be used as a source of nitrogen (**N**) for microbial synthesis, increasing the supply of N to the rumen in low-protein diets, and has a lower cost of N relative to other protein feeds (Leng and Nolan, 1984; Leng, 2008). Urea is the most common source of NPN and has been included in ruminant diets as a source of N or to increase rumen degradable protein (Leng and Nolan, 1984). Urea is rapidly hydrolyzed to carbon dioxide and ammonia through rumen microbial ureases (Nichols et al., 2022). Other NPN sources, such as nitrates and biuret, have also been evaluated in ruminant diets. In the rumen, nitrates are reduced to nitrites and ammonia, competing for reducing equivalents with rumen methanogenesis and decreasing methane emissions (Ungerfeld, 2015; Honan et al., 2022). Nitrate reduction occurs rapidly in the rumen and requires an adaptation period to increase nitrate–nitrite reductive bacteria to avoid nitrite accumulation (Leng, 2008; Lee and Beauchemin, 2014). Nitrites are absorbed through the rumen wall and may impair oxygen transportation due to methemoglobin formation (Lee and Beauchemin, 2014). Biuret is slowly hydrolyzed to carbon dioxide and ammonia in the rumen and requires microbial adaptation to synthesize biuretases by ruminal microorganisms (Shirley, 1986). Thus, ammonia is the expected product of hydrolysis or reduction of different NPN sources in the rumen.

Ruminal fermentation requires available energy and N for volatile fatty acid (**VFA**) and microbial synthesis (Ungerfeld and Hackmann, 2020). However, the hydrolysis and reduction of different NPN sources should result in different ruminal fermentation profiles. For example, ammonia concentration peaks rapidly after the consumption of urea (Nichols et al., 2022). Nitrates show a delayed peak in ammonia concentration relative to urea because they require the reduction of nitrates to nitrites and then to ammonia (Leng, 2008; El-Zaiat et al., 2014). Furthermore, biuret supplementation does not show an ammonia peak due to its slow solubility (Bartle et al., 1998). Thus, it is expected that the energy required for microbial synthesis should be different when different sources of NPN are provided. It is possible to include a mixture of different NPN sources to provide N at different time points, increasing the fermentation and microbial synthesis due to the synchronization of N and energy. However, there is scarce information regarding the effect of different NPN mixtures on fermentation profile, methane production, and microbial synthesis (Löest et al., 2011).

This experiment aimed to evaluate the effect of different NPN sources and their combinations on fermentation profile, methane production, and microbial N synthesis in an in vitro system using a backgrounding substrate. The experimental hypotheses were 1) supplementation of NPN would increase ammonia concentration, promoting greater rumen microbial fermentation, and resulting in greater organic matter digestibility (**OMD**), and 2) increasing nitrate proportion in the NPN mixture would decrease methane production because nitrates negatively affect the methanogenesis.

## MATERIALS AND METHODS

This study was conducted at the North Florida Research and Education Center (NFREC) in Marianna, FL. All procedures involving animals were approved by the University of Florida Institutional Animal Care and Use Committee (#202111460).

### Animal Management and Diet Adaptation

Two ruminally cannulated Angus crossbred steers (808 ± 36.3 kg of body weight) were used as ruminal fluid donors for the in vitro batch culture incubations. The steers were fed a diet comprised of corn silage, cotton gin byproduct, and a premix of vitamins and minerals (70, 28, and 2% on a dry matter basis, respectively) ad libitum at least 35 d before collecting ruminal fluid to perform the in vitro incubations. Each steer received an NPN mixture equivalent to 100 g of urea/d (i.e., 46 g of N daily/steer) in the diet comprised of an equal amount of N from the different NPN sources to adapt the rumen microbial community. Thus, each steer was fed daily 33 g of urea (46% N, Yara International, Oslo, Norway), 37 g of a urea–biuret mixture (41% N, Yara International), and 97 g of calcium–ammonium nitrate (15.5% N, Yara International). The nitrate source was gradually introduced to the diet as follows: 30% of the total final amount during the first week (i.e., 29 g/steer/day), 60% of the total final amount during the second week (i.e., 58 g/steer/d), and 100% of the total final amount during the third week until the end of the experiment (i.e., 97 g/steer/d).

### Experimental Design and Dietary Treatments

In vitro incubations were conducted on three separate days (replicates) using the same corn silage, cotton gin byproduct, and NPN sources fed to the steers as an incubation substrate. Corn silage and cotton gin byproduct were used as a substrate at 70% and 30% of dry matter (**DM**), respectively. Dietary treatments were as follows: control without NPN supplementation (**CON**), 100% urea (**UR**, Rumisan, Yara International), and five different proportions (100:0, 75:25, 50:50, 25:75, and 0:100) of urea–biuret and nitrates (**UBN**, Bolifor, Yara International). Treatments were designed to be isonitrogenous and equivalent to 1% of the inclusion of urea in the diet DM, except for the CON treatment.

A representative sample of digesta was collected from different places in the rumen from two ruminally cannulated Angus crossbred steers and strained through four layers of cheesecloth, placed in prewarmed thermos containers, and transported to the laboratory within 30 min of collection. In the laboratory, ruminal fluid from the two steers was maintained under constant CO_2_ flux and was combined in equal proportions. A 4:1 McDougall’s buffer–ruminal fluid mixture was used for all incubations (McDougall, 1948). McDougall’s buffer was mixed with and without 0.077 g/L of ammonium sulfate 10% enriched with ^15^N.

Two 500-mL bottles fitted with a side arm and a rubber septum were incubated per treatment, and one 500-mL bottle as a blank. Bottles containing 5.6 g of the substrate and 400 mL of ^15^N enriched inoculum were incubated for 24 h at 39 °C with gentle agitation (60 rpm) to monitor gas production kinetics using the Ankom Gas Monitoring System (Ankom Technologies, Macedon, NY). Also, one 500-mL bottle without ^15^N enriched inoculum was incubated to determine the basal ^15^N enrichment. At 12 h of incubation, two 10-mL rumen fluid samples were collected through the septa port using a 20-mL syringe. Samples were acidified by adding 10 µL of a 20% (vol/vol) H_2_SO_4_ solution to each 10-mL sample and were frozen at −20 °C until further analysis. At the end of the 24 h incubation, the final pH was recorded, bottles were placed on ice to prevent further microbial fermentation, and two 10-mL samples were collected and acidified by adding 10 µL of a 20% (vol/vol) of H_2_SO_4_ solution and were frozen at −20 °C until further analysis. Then, the remaining fermentation content was agitated (60 rpm) for 30 s, divided into two equal parts (i.e., whole content and the bacterial pellet with approximately 180 mL each), and preserved at −20°C until further analysis.

Additionally, two tubes of 100 mL per treatment containing 0.7 g of the treatment substrate and 50 mL of inoculum (without ^15^N enrichment) and two blanks were incubated for 24 h at 39 °C with gentle agitation (60 rpm). After the 24-h incubation with ruminal fluid, 6 mL of HCl and 2 mL of a 5% (wt/vol) pepsin (1:3,000; Amresco Inc., Solon, OH) solution were added. Tubes were incubated for another 48 h at 39 °C. After the incubation period, tubes were maintained on ice to prevent further microbial fermentation, and the content was filtered (Fisherbrand Filter Paper P8; Fisher Scientific, Pittsburg, PA) for further analysis.

### Gas and Methane Production

Cumulative gas production was recorded using the Ankom Gas Monitoring System (Ankom Technologies) during the entire fermentation process. The kinetics of gas production was fitted to the Gompertz model to determine the rate of total maximal gas production (M), fractional rate of gas produced (kf), and lag phase (L; Schofield et al., 2018). Total gas produced in the 500-mL bottles was collected in a 1-L Tedlar gas collection bag (Supelco Analytical, Bellefonte, PA) attached to the Ankom Gas Monitoring modules. A subsample of the gas in the Tedlar bag was collected and analyzed for methane concentration.

### Microbial Protein Synthesis

Total N and isotopic composition were measured in the two probes (i.e., whole content and the bacterial pellet) by isotopic ratio mass spectrometry (Vario Micro cube, Elementar Analyzer system GmbH., Langenselbold, Germany). The bacteria pellet subsample probe was slowly centrifuged at 1,000 × *g* for 10 min at 4 °C, and the resulting supernatant was centrifuged at 20,000 × *g* for 20 min at 4 °C to obtain the pellet of bacteria. The bacteria pellet was washed with 0.9% (wt/vol) saline solution and centrifuged at 20,000 × *g* for 20 min at 4 °C, discarding the supernatant. The saline wash procedure was repeated twice. Finally, the bacteria pellet was freeze-dried. The whole content probe was completely freeze-dried. Dried samples of whole content and bacterial pellet were manually grounded and weighted into an 8 × 5-mm pressed standard-weight tin capsule (Elemental Microanalysis, Okehampton, UK) using a Mettler-Toledo Excellence Plus XP Micro Balance (Mettler-Toledo GmbH, Laboratory and Weighing Technologies, Greifensee, Switzerland). Then, samples were preserved at room temperature for further analysis.

### Laboratory Analyses

Corn silage and cotton gin byproduct samples were analyzed by a commercial laboratory using a wet chemistry package for N, neutral detergent fiber, acid detergent fiber, and ash (Dairy One, Ithaca, NY; Table 1). Further, approximately 0.5 g of sample were weighed in duplicate, dried in a forced air oven at 100 °C for 24 h, and ashed at 550 °C for 6 h to determine the DM and organic matter (**OM**) of the NPN sources. Also, the concentration of N was analyzed through the Dumas dry combustion method after samples were ball-milled using a Mixer Mill MM400 (Retsch) at 25 Hz for 9 min using a Vario Micro Cube (Elementar, Manchester, UK; Table 1).

**Table 1.** Chemical composition (% DM) of the ingredients used in the experiment

Chemical analyses^1^	Ingredient				
	Corn silage	Cotton gin byproduct	Urea^2^	Urea–biuret mixture^3^	Nitrates^4^
N	1.3	2.6	46.0	41.0	15.5
NDF	27.4	61.7	—	—	—
ADF	16.6	58.8	—	—	—
Ash	2.8	10.3	0.2	0.4	32.8
OM	97.2	89.7	99.8	99.5	67.2

^1^N: nitrogen; NDF: neutral detergent fiber; ADF: acid detergent fiber; OM: organic matter (OM = 100 − ash [%]).

^2^Rumisan (Yara International, Oslo, Norway).

^3^Urea–biuret mixture (Yara International).

^4^Bolifor (Yara International).

Preserved fermentation residues from 100 mL tubes were filtered and dried at 105 °C in a forced air oven for 24 h and ashing at 550 °C for 6 h to determine the undigested dry and OM, respectively.

The concentration of VFA at 12 and 24 h was determined in a liquid–liquid solvent extraction using ethyl acetate (Ruiz-Moreno et al., 2015). Samples were centrifuged for 15 min at 10,000 × g. Ruminal fluid supernatant was mixed with a *meta*-phosphoric acid (25%, wt/vol): crotonic acid (2 g/L, internal standard) solution at a 5:1 ratio, and samples were frozen overnight, thawed, and centrifuged for 10 min at 10,000 × *g*. The supernatant was transferred into glass tubes (12 mm × 75 mm; Fisherbrand; Thermo Fisher Scientific Inc., Waltham, MA) and mixed with ethyl acetate in a 2:1 ratio of ethyl acetate to the supernatant. After shaking tubes vigorously and allowing the fractions to separate, the ethyl acetate fraction (top layer) was transferred to vials (9 mm; Fisherbrand; Thermo Fisher Scientific Inc.) Samples were analyzed by gas chromatography (Agilent 7820A GC, Agilent Technologies, Palo Alto, CA) using a flame ionization detector and a capillary column (CP-WAX 58 FFAP 25 m × 0.53 mm, Varian CP7767, Varian Analytical Instruments, Walnut Creek, CA). The column temperature was maintained at 110 °C, and injector and detector temperatures were 200 and 220 °C, respectively.

The concentration of NH_3_-N at 12 and 24 h was analyzed after centrifuging ruminal fluid samples at 10,000 × *g* for 15 min at 4 °C (Avanti J-E, Beckman Coulter Inc., Palo Alto, CA) following the phenol-hypochlorite technique described by Broderick and Kang (1980) with the following modification: absorbance was read on 200 µL samples at OD620 in flat-bottom 96-well plates (Corning Costar 3361, Thermo Fisher Scientific Inc.) using a plate reader (Fisherbrand UV/VIS AccuSkan GO Spectrophotometer, Thermo Fisher Scientific Inc.).

The concentration of CH_4_ was determined using a gas chromatograph (Agilent 7820A GC, Agilent Technologies) with flame ionization and a capillary column (Plot Fused Silica 25 m × 0.32 mm, Coating Molsieve 5A, Varian CP7536, Varian Inc.) The injector, column, and detector temperatures were 80, 160, and 200 °C, respectively, and N_2_ was the carrier gas flowing at 3.3 mL/min. The split ratio for the injected CH_4_ sample was 100:1.

Whole content and bacteria pellet samples received 35 μL of a 10 g/L solution of K_2_CO_3_ and were dried overnight in a forced air oven at 60 °C for complete NH_3_-N evaporation. The N content in the whole content after NH_3_-N volatilization is considered non-ammonia nitrogen. Finally, the concentration of N and percentage of atom ^15^N in dried samples was analyzed in an isotope ratio mass spectrometer (IsoPrime 100, IsoPrime, Manchester, UK).

### Calculation and Statistical Analysis

The in vitro dry matter digestibility (**DMD)** and OMD were calculated as follows:


$$\begin{array}{l} IVDMD(\%)=100\times \left(\frac{Incubated\;DM-residual\;DM}{\;Incubated\;DM}\right), \end{array}$$

$$\begin{array}{l} IVOMD(\%)=100\times \left(\frac{Incubated\;OM-residual\;OM}{\;Incubated\;OM}\right). \end{array}$$

Also, microbial N synthesis was determined according to Bach and Stern (1999) as follows:


$$\begin{array}{l} Microbial\;N(g)=NAN(g)\times \left(\frac{\%atom\;excess\;of\;15N\;in\;the\;whole\;content}{\;\%atom\;excess\;of\;15N\;in\;the\;microbial\;pellet}\right). \end{array}$$

Moreover, indicators of metabolization of N and microbial efficiency were determined according to Bach and Stern (1999) as follows:


$$\begin{array}{l} Microbial\;ef\;f\;iciency\;(ME)=\left(\frac{Microbial\;N(g)}{\;OM\;truly\;digested(kg)}\right), \end{array}$$

$$\begin{array}{l} Ef\;f\;iciency\;of\;N\;use(ENU)=\left(\frac{Microbial\;N(g)}{N\;incubated(g)}\right). \end{array}$$

Organic matter truly digested was calculated as the difference between the amount of OM incubated and the amount of OM digested corrected by the amount of microbial OM, as adapted from Soder et al. (2013). Microbial OM was calculated as the DM of the bacterial pellet corrected by the ruminal microbial OM concentration (Czerkawski, 1978).

Data were analyzed as a randomized complete block design with three replicates (blocks) using the MIXED procedure of SAS (SAS Institute Inc., Cary, NC). The average of two bottles or tubes within each incubation day was considered the experimental unit, and the model included the fixed effects of NPN treatment and the random effect of incubation day (replicate). Orthogonal contrasts were used to partition specific treatment effects. Contrasts were 1) CON vs. NPN supplementation, 2) UR vs. UBN mixtures, and 3) linear, 4) quadratic, 5) cubic, and 6) quartic responses of the different proportions of UBN mixtures. Linear response was only significant among UBN mixtures. Significance was declared at *P* ≤ 0.05, and tendencies were considered when 0.10 > *P* > 0.05.

## RESULTS

### Non-Protein Nitrogen Supplementation on In Vitro Fermentation Profile and Digestibility

At 12 h of fermentation, NPN supplementation increased (*P* < 0.05) the concentration of the total VFA, acetate, and NH_3_-N and tended (*P* = 0.054) to increase the concentration of butyrate. Also, NPN supplementation increased (*P* < 0.05) the proportion of acetate and tended (*P* = 0.054) to reduce the proportion of propionate resulting in a greater (*P* < 0.05) acetate–propionate ratio. Urea supplementation showed a lower (*P* < 0.01) proportion of acetate and tended (*P* = 0.06) to show a lower concentration of acetate and proportion of butyrate relative to the UBN treatments. Increasing the proportion of nitrates in the UBN mixture increased (*P* < 0.001) the proportion of acetate and reduced (*P* < 0.01) the concentration and the proportion of butyrate (Table 2).

**Table 2.** Effect of inclusion and source of NPN^1^ on the concentration of VFA and NH_3_-N at 12 h of incubation in a corn silage-based diet

Variable	CON	UR	UBN mixtures					SEM^2^	*P*-value		
			100:0	75:25	50:50	25:75	0:100		CON vs. NPN	UR vs. UBN	Linear response of UBN mixtures
Total VFA, mM	30.95	31.77	32.55	32.92	32.13	33.53	32.06	1.035	0.049	0.248	0.862
Acetate, mM	16.79	17.65	17.29	18.28	18.02	19.01	18.24	0.587	0.019	0.060	0.191
Propionate, mM	8.79	8.72	8.85	8.77	8.56	8.82	8.54	0.803	0.727	0.968	0.436
Butyrate, mM	4.17	4.64	4.80	4.70	4.40	4.49	4.16	0.543	0.054	0.467	0.010
Acetate, mol/100 mol	54.28	54.30	54.24	55.52	56.07	56.65	56.90	0.539	0.005	0.002	0.001
Propionate, mol/100 mol	28.35	27.38	27.11	26.57	26.68	26.19	26.56	1.964	0.054	0.336	0.514
Butyrate, mol/100 mol	13.55	14.65	14.82	14.34	13.68	13.54	13.02	1.917	0.221	0.055	0.001
Acetate–propionate	1.93	2.00	2.03	2.11	2.17	2.18	2.17	0.166	0.040	0.122	0.139
NH_3_-N, mM	0.35	2.74	2.61	2.72	2.31	2.44	2.11	0.139	0.001	0.573	0.405

^1^CON: control without NPN supplementation; UR: urea supplementation; UBN: urea–biuret and nitrates mixture supplementation.

^2^SEM: pooled standard error of the treatment means, *n* = 3/treatment.

At 24 h of fermentation, treatments did not modify (*P* > 0.05) the total concentration of VFA. However, NPN supplementation increased (*P* < 0.05) the concentration of ammonia and the proportion of acetate, resulting in a tendency (*P* = 0.076) to increase the acetate–propionate ratio. Urea inclusion reduced (*P* < 0.05) the concentration and proportion of acetate relative to the UBN mixtures. Increasing the proportion of nitrate in the UBN mixture increased (*P* < 0.01) the proportion of acetate, reduced (*P* < 0.05) the proportion of butyrate, and tended to reduce (*P* = 0.09) the concentration of NH_3_-N. The final pH and the digestibility of dry and OM did not differ (*P* > 0.05) among treatments (Table 3).

**Table 3.** Effect of inclusion and source of NPN^1^ on pH, VFA and NH_3_-N concentration, and digestibility at 24 h of incubation in a corn silage-based diet

Variable	CON	UR	UBN mixtures					SEM^2^	*P*-value		
									CON vs. NPN	UR vs. UBN	Linear response of UBN mixtures
			100:0	75:25	50:50	25:75	0:100				
pH	6.34	6.38	6.37	6.36	6.35	6.39	6.37	0.001	0.122	0.491	0.584
Total VFA, mM	41.39	40.96	42.85	43.58	42.86	43.50	42.14	1.837	0.302	0.113	0.671
Acetate, mM	22.17	21.94	23.03	24.14	23.53	24.24	23.47	1.201	0.144	0.049	0.672
Propionate, mM	11.55	11.23	11.57	11.48	11.45	11.42	11.29	0.997	0.571	0.415	0.425
Butyrate, mM	5.91	5.98	6.35	6.13	6.05	5.92	5.96	0.585	0.780	0.922	0.103
Acetate, mol/100 mol	53.47	53.52	53.72	55.61	54.84	55.64	55.67	0.544	0.012	0.005	0.007
Propionate, mol/100 mol	27.88	27.30	26.94	26.33	26.74	26.18	26.73	1.594	0.181	0.408	0.814
Butyrate, mol/100 mol	14.41	14.78	14.91	14.17	14.14	13.80	13.37	1.792	0.690	0.218	0.047
Acetate–propionate	1.92	1.97	2.01	2.11	2.09	2.13	2.10	0.120	0.076	0.152	0.384
NH_3_-N, mM	2.48	6.55	7.24	7.62	6.34	6.55	4.96	0.283	0.002	0.999	0.091
IVDMD^3^, %	45.02	45.08	45.78	48.35	47.55	46.60	51.08	1.031	0.542	0.483	0.442
IVOMD^4^, %	64.13	65.38	64.17	65.47	65.66	65.18	67.96	0.403	0.863	0.917	0.404

^1^CON: control without NPN supplementation; UR: urea supplementation; UBN: urea–biuret and nitrates mixture supplementation.

^2^SEM: pooled standard error of the treatment means, *n* = 3/treatment.

^3^In vitro dry matter digestibility.

^4^In vitro organic matter digestibility.

### Non-Protein Nitrogen Supplementation on Microbial N Synthesis, Gas, and Methane Production

The supplementation of NPN increased (*P* < 0.05) the microbial N synthesis and tended to increase (*P* = 0.083) the bacteria efficiency. Urea supplementation did not differ (*P* > 0.05) from UBN combinations on microbial N synthesis. However, increasing the proportion of nitrates in the UBN mixture linearly increased (*P* < 0.05) the microbial N synthesis and the efficiency of N use (Table 4).

**Table 4.** Effect of inclusion and source of NPN^1^ on the microbial nitrogen synthesis and microbial efficiency production in a corn silage-based diet

Variable	CON	UR	UBN mixtures					SEM^2^	*P*-value		
									CON vs. NPN	UR vs. UBN	Linear response of UBN mixtures
			100:0	75:25	50:50	25:75	0:100				
Microbial N, mg	55.66	67.34	59.55	62.58	68.70	64.68	70.88	3.584	0.006	0.511	0.016
BE^3^, g/kg	27.07	34.46	41.98	35.18	37.39	36.12	39.05	4.945	0.083	0.541	0.762
ENU^4^, g/g	57.16	55.00	48.57	51.63	57.24	54.44	60.28	2.995	0.308	0.825	0.004

^1^CON: control without NPN supplementation; UR: urea supplementation; UBN: urea–biuret and nitrates mixture supplementation.

^2^SEM: pooled standard error of the treatment means, *n* = 3/treatment.

^3^Bacteria efficiency = bacteria N (g)/OM truly digested (kg).

^4^Efficiency of N use = (bacterial N [g]/N incubated [g]) × 100.

Total gas and methane production did not differ (*P* > 0.05) among NPN supplementation. However, NPN supplementation reduced the phase lag time (i.e., L) and the fractionate rate of gas production (i.e., kf) relative to the control treatment. Additionally, increasing the proportion of nitrate in the UBN mixture reduced (*P* < 0.05) the CH_4_ production per unit of dry incubated or OM digested (Table 5).

**Table 5.** Effect of inclusion and source of NPN^1^ on gas and methane production in a corn silage-based diet

Variable^2^	CON	UR	UBN mixtures					SEM^3^	*P*-value		
			100:0	75:25	50:50	25:75	0:100		CON vs. NPN	UR vs. UBN	Linear response of UBN mixtures
M, mL/g OMi	251.7	243.5	233.5	224.8	236.4	212.7	232.0	5.575	0.326	0.472	0.822
M, mL/g OMd	277.8	268.4	263.8	244.0	260.7	233.1	255.1	5.682	0.286	0.444	0.682
L, h	0.13	0.58	0.81	0.84	1.07	1.52	1.23	0.080	0.014	0.120	0.144
kf, %/h	6.30	8.43	8.40	9.13	8.29	9.38	7.35	0.27	0.049	0.943	0.577
Methane, mM/g OMi	1.24	1.23	1.18	1.16	1.12	1.07	0.83	0.025	0.142	0.118	0.016
Methane, mM/g OMd	6.93	6.86	6.58	6.46	6.25	5.96	4.65	0.142	0.153	0.127	0.017

^1^CON: control without NPN supplementation; UR: urea supplementation; UBN: urea–biuret and nitrates mixture supplementation.

^2^M: maximal gas production; L: lag phase of gas production; kf: fractional rate of gas production; OMi: organic matter incubated; OMd: organic matter digested.

^3^SEM: pooled standard error of the treatment means, *n* = 3/treatment.

## DISCUSSION

Dietary supplementation of NPN increases ruminal fermentation and microbial synthesis when ruminants are fed diets with decreased crude protein concentration (Currier et al., 2004). In this experiment, adding NPN increased the NH_3_-N concentration at 12 and 24 h of incubation. Additionally, the concentration of total VFA was increased at 12 h but not at 24 h of incubation (Tables 2 and 3). The increase in the concentration of total VFA at 12 h was largely driven by an increase in the concentration of acetate (Table 2). These results are in line with previous studies showing that increased NPN supplementation increases NH_3_-N availability in vitro and in vivo (Patra and Yu, 2015; Henry et al., 2020), possibly generating greater fibrolytic activity and thereby increasing the molar proportion of acetate and the acetate–propionate ratio during the fermentation (Russell et al., 1992). The digestibility of OM increased when NPN was supplemented in a forage-based substrate (Adejoro and Hassen, 2018; Henry et al., 2021). However, total gas production showed inconsistent results in previously published in vitro studies. Henry et al. (2021) reported an increase in total gas production, while Adejoro and Hassen (2018) did not find differences when urea or nitrates were supplemented under in vitro conditions. In this experiment, supplementation of NPN did not change the digestibility of DM and OM or the maximal gas production after 24 h of incubation (Tables 3 and 5). Additionally, NPN supplementation increased L and kf parameters relative to CON (Table 5), indicating that although the ruminal microbial community required more time to start the fermentation, the fermentation dynamic was faster, achieving similar conditions by the end of the incubation. These observations agree with the greater concentration of total VFA at 12 h when including NPN supplementation, and not at 24 h when total VFA concentrations do not differ among treatments (Tables 2 and 3). As mentioned, supplementation with NPN could promote fibrolytic bacteria, explaining the greater L and kf parameters. The establishment of fibrolytic community is slow (i.e., greater L; Shirley, 1986), and possibly the greater NH_3_-N availability during the fermentation when NPN was supplemented allowed faster digestibility (i.e., greater kf) and greater microbial N synthesis (Table 4). However, the digestibility of DM and OM were similar after 24 h of incubation, meaning that either the remaining substrate contained a similar undegradable fraction or the microbial activity was reduced at the late fermentation stage, resulting in a similar final fermentation profile among treatments. Given that NH_3_-N concentrations at 12 and 24 h are greater for NPN-supplemented treatments, the availability of N may not explain differences in microbial activity at later stages of the fermentation. Additionally, in vitro batch culture systems have limitations that may affect the fermentation dynamic and microbial communities (López, 2005), such as the accumulation of end products (e.g., VFA and NH_3_-N) or the impossibility to evaluate the fermentation turnover (e.g., passage rate or VFA absorption). All sources of NPN showed similar fermentation profiles at 24 h of incubation (Table 3). Biuret and nitrates supplementation require an adaptation period to acclimate the rumen microbial communities differently than urea (Schröder and Gilchrist, 1969; Lee and Beauchemin, 2014). The adaptation period of biuret varies between 14 and 70 d (Farlin et al., 1968; Oltjen et al., 1969), while nitrates seem to require at least 21 d of adaptation (Leng, 2008), although it is expected to vary among animals. Biuret and nitrate inclusion possibly reduced the digestibility in other in vitro studies because the ruminal microbiota was not adequately adapted (Belasco, 1954; Henry et al., 2021). In this experiment, donor animals were adapted during 35 d to the different sources of NPN, showing a similar fermentation profile and suggesting an adequate adaptation period.

Increasing nitrates proportion in the UBN mixture increased the proportion of acetate and reduced the proportion of butyrate at 12 and 24 h of incubation (Tables 2 and 3). Similarly, nitrate supplementation increased the acetate proportion and reduced the propionate and butyrate proportion using a forage-based substrate (Adejoro and Hassen, 2018). Nitrate supplementation modifies ruminal fermentation because it can capture hydrogen (H_2_) during the reduction to ammonia. Lower H_2_ pressure in the rumen promotes the synthesis of oxidized products such as acetate and decreases the synthesis of more reduced compounds such as propionate or butyrate (Janssen, 2010; Ungerfeld, 2020; van Lingen et al., 2021). Furthermore, the reallocation of H_2_ competes with methanogenesis during nitrate reduction (Ungerfeld, 2015). Additionally, nitrates supplementation might reduce methane production because some intermediate compounds produced during the nitrate reduction to ammonia, like nitrite or nitrous oxide, show antimicrobial effects affecting methanogenic populations (Marais et al., 1988; Leng, 2008; Granja-Salcedo et al., 2019). Therefore, nitrate supplementation is associated with lower CH_4_ production in vitro and in vivo (Božic et al., 2009; Feng et al., 2020; Fouts et al., 2022). In this experiment, CH_4_ production was decreased linearly with increasing the proportion of nitrates in the UBN mixture (Table 5).

Supplementation of NPN increased microbial N synthesis and tended to increase the ME after 24 h of incubation (Table 4). As mentioned, NPN supplementation provides N for ruminal bacteria in diets with lesser protein concentration, especially fibrolytic microorganisms (Fonnesbeck et al., 1975; Leng, 2008). In this experiment, NPN supplementation increased the concentration of total VFA at 12 h and kf, suggesting greater digestibility at the early stages of the fermentation. Thus, greater microbial N could have been produced at the beginning of the fermentation due to the greater concentration of available NH_3_-N in the incubation fluid (Tables 2 and 3). Additionally, increasing the concentration of nitrates in the UBN increased microbial N synthesis and EUN (Table 4). This result agrees with the tendency to reduce NH_3_-N when including nitrates in the UBN mixture (Table 3). Differences in the availability of energy and N and the reallocation of fermentation routes due to greater H_2_ capture would promote greater microbial synthesis when nitrates are included (Ungerfeld and Hackmann, 2020).

In conclusion, NPN supplementation increased the concentration of NH_3_-N and modified the gas production dynamic, suggesting greater microbial activity at the initial stages of the fermentation and resulting in greater microbial N synthesis. Even though NPN sources did not modify digestibility and total gas production, increasing the proportion of supplemental nitrates reduced CH_4_ production and increased the molar proportion of acetate and the microbial N synthesis. These results prove that it is possible to formulate NPN sources, including urea, biuret, and nitrate combinations modifying the fermentation profiles and potentially reducing methane compared to including urea only. Future research with in vivo conditions is necessary to validate the conclusions that can be drawn from this study.
